# Improving the Safety Performance of Construction Workers through Individual Perception and Organizational Collectivity: A Contrastive Research between Mainland China and Hong Kong

**DOI:** 10.3390/ijerph192114599

**Published:** 2022-11-07

**Authors:** Xiangcheng Meng, Alan H. S. Chan

**Affiliations:** 1 Sub-Institute of Public Security, China National Institute of Standardization, Beijing 100191, China; 2 Department of Advanced Design and Systems Engineering, City University of Hong Kong, Hong Kong 999077, China

**Keywords:** safety performance, individual perception, organizational collectivity, comparative study

## Abstract

Considering the increasing number in construction accidents in Mainland China and Hong Kong, research on improving the safety performance of construction personnel is important, given the essential role it plays in occupational safety development in industries. The present study aimed to assess the improving channels of safety performance through individual perception and organizational collectivity in a quantitative way by integrating safety motivation as the transition role between individual and organizational levels. The questionnaire survey was conducted with 180 participants from Hong Kong and 197 responses from Mainland China. Structural equation modeling was applied to investigate and compare the direct, indirect, and mediating effects among different safety constructs. This study is unique, as it firstly integrates the theories of personal cognition and group interaction together with the mechanism of safety performance development. Such integration can increase the effectiveness of reducing the unsafety of construction workers at both individual and organizational levels, thereby reducing the numbers of construction accidents, and promoting healthy occupational development of the personnel.

## 1. Introduction

The construction industry plays a significant role in the growth of global economic and workforce employment. However, personnel injuries and safety accidents still frequently occur, which have caused huge costs in personal, social, and financial aspects in global range [1]. For instance, Mainland China had 2485 accidents and 2851 deaths on average each year from 2009 to 2018 in a building site. Specifically, the number of deaths from construction accidents in 2018 was 3952, which has a 30.8% upward tendency compared with that of 2017 [2]. A similar situation was observed in Hong Kong, which is one of the special administrative regions in China. The large amount of labor force employment was provoked by the flourishing of the construction industry in recent years, followed by a certain degree of high mortality. According to the Hong Kong Labor Department, 3541 local construction accidents occurred in 2018, while the accident rate per 1000 construction workers decreased by only 3.7% from 35.4% to 31.7% [3]. These statistics indicate an urgent need to reduce accidents that must be emphasized seriously by all scholars and industrial personnel concerned with the occupational health and safety of the construction industry, in both Hong Kong and Mainland China.

According to the previous findings of construction safety research, safety performance (SP) has been adopted as a practical indicator to present the safety status of construction personnel instead of using statistical data of casualties and fatalities [4]. Therefore, the analysis of modeling SP improvement by integrating promising theories of the safety constructs is meaningful and contributive for occupational safety development of the building industry [5]. Researchers previously considered the SP mechanism as an individual construct influenced by individual perception and personal characteristics, such as personal attitude, cognition, and mentality [6,7]. However, the mechanism of SP should not be isolated at a personal level, since the negative organizational issues, such as a poor organizational atmosphere and culture, also play a role in causing unsafe performance during the working process [8,9]. Therefore, researchers have encouraged the analysis of SP mechanism as the integration of organizational interaction and individual cognition [10].

This study integrates two types of SP models (individual based model and organizational based model) together by linking them with a potential correlation among individual risk perception and organizational collectivity [11,12], thus achieving a dual perspective on occupational safety improvement of construction personnel. A comparative study was conducted between Mainland China and Hong Kong to reveal their regional similarities and differences due to subtle cultural and social distinctions [13].

The aims of this study involve the following:

(1) Offering a theoretical foundation in influence path of individual perception and organizational collectivity on SP.
(2) Comparing the similarities and differences in the influencing mechanism on SP between Mainland China and Hong Kong to provide targeted safety suggestions.

## 2. Materials and Methods

### 2.1. Safety Performance

As a practical indicator of personnel safety, safety performance (SP) is defined as the individual compliance and organizational participation that can contribute to working safety and further classified into two dimensions: safety participation (SP1) and safety compliance (SP2) [4,14]. As defined, safety compliance involves personal adherence to safety regulation and standard procedures, as well as the obedience in safety work manner in individual perspectives, while safety participation is defined as voluntary work with the aim of providing safety support among group members and promoting safety in an organizational aspect [15,16].

SP is usually considered to be influenced by personal factors, such as safety knowledge and safety investment [7,17]. However, the mechanism of SP should not be isolated at the individual level. The research reveals that organizational issues also play a role in causing unsafe performance of personnel during the working process instead of the individual violation of workers unilaterally [8]. Therefore, researchers encourage modeling the influence mechanism of SP as the integration of both individual and organizational levels [3].

### 2.2. Individual and Organizational Influence

#### 2.2.1. Individual Perception Mechanism

Xia et al. established a cognitional model at the individual level to verify the influence mechanism of workers’ SP, which involves risk perception as influential factors [12]. RPC refers to personnel’s self-judgement toward the “probability” of being in an accident, the “seriousness” of the potential consequence, and whether they feel “unsafe” in terms of the outcomes of dangerous scenes [18]. Various efforts of previous studies were made for the understanding of RPC effect on human safety. Plapp and Werner identified RPC as the fundamental element for performance toward risks response and natural hazards [19]. The research scope was further extended to involve public health-related issues [20] and technological hazards [21]. For occupational safety in building construction, the positive effect of RPC was revealed towards various safety performance of workers, such as the compliance of using protective equipment [22] and participation in safety management training [23].

#### 2.2.2. Organizational Collectivity Mechanism

With regard to the organizational aspect, the previous studies demonstrated the possible improvement of SP through the interaction among organizational members, including group cohesion and collective efficiency [11,24]. The former refers to the relation of harmony and cooperation among organizational members [25,26]. The latter is recognized as members’ judgment of group ability or the evaluation of collective ability to complete upcoming work, which represents the outcome of an internal process of the correlational improvement in the organization [27,28]. Both constructs were defined as components of organizational collectivity, with (OC) considering the decisive conditions involved in the OC [29], namely, a formal work organization characterized by efficiency and profitability, as well as the harmonious employment relationship between employee and leaders [30]. As defined, OC helps foster the occupational safety of the workers since the safety performance is achieved through an unwavering dedication and commitment of group members from all levels in the organization [31,32].

### 2.3. Safety Motivation

As defined, safety motivation (SMO) refers to the willingness and intentions of the personnel to exert effort for enacting safety behaviors and the relevant behavioral valence [33,34]. The causality exists between safety motivation and workers’ behavior in construction workplace, as a stronger safety motivation among employees leads to higher probability of conducting safe behavior practices [35]. Thus, safety motivation among the leader and subordinate is essential to create a safe workplace with few accidents [36]. In addition, the absence of SMO when implementing health and safety promotion at a construction industry has been considered a major reason for unsafe performance of personnel, such as the violation of regulations and less involvement of workplace safety education [37].

SMO can be positively influenced by individual RPC as the perceptions of workplace risks likely influence workers’ motivation to act safely first and then promote their safety behavior [38]. Furthermore, SMO and RPC were demonstrated to be the influential factors of workers’ behavioral participance and compliance towards safety [39,40].

For an organizational SP model, the workers from a cohesive group usually show high willingness to participate in safety contribution and obey the requirement of their supervisor, thereby achieving better performance in work safety compared with the disunited groups [41]. In addition, the efficiency of collectiveness motivates a positive atmosphere of employee participance and obedience [42]. According to Lewis (2011), high level of OC will motivate the personnel engagement towards work and the organization [43], as well as encourage the willingness of group members to exert effort at safety improvement [12].

### 2.4. Regional Comparison

One of the research objectives is to carry out the contrastive study between Mainland China and Hong Kong. A subtle relationship between these two regions has existed for many years. For political and cultural aspects, an abstractly detached sense of traditional Chinese culture is identified in Hong Kong, though the administrative pattern (capitalist system) is distinguished with the communism applied in Mainland China [44]. For the social development aspect, Hong Kong government incorporated itself with local and overseas elites into the administration and political consultation at the beginning of its development stage, which can be mainly attributed to its advanced international background [13]. However, after 1979, the implementation of the reform and opening-up policy has accelerated the development pace of social economy and technology in Mainland China [45]. Even after numerous years of changes in the two regions, personnel safety in the construction industry has been considered an important issue in Hong Kong and Mainland China [46,47]. However, no current studies have focused on the discovery of the influence mechanism in the SP of construction personnel integrating the safety constructs at both individual and organizational levels, let alone the regional comparison between Mainland China and Hong Kong. Therefore, in this study, territorial similarities and differences of the influence mechanism of SP are investigated between the two regions by collecting research data from 180 Hong Kong respondents and 197 Mainland participants.

### 2.5. Hypotheses

The present study postulated five hypotheses to verify the corresponding direct correlations between factors in the research model of safety performance. Seven hypotheses were put forwards in line with the correlation potentiality among individual perception, organizational collectivity, and safety performance of the personnel.

**Hypothesis** **1.**  *RPC significantly and positively influences SP.*

**Hypothesis** **2.**  *RPC significantly and positively influences SMO.*

**Hypothesis** **3.**  *SMO significantly and positively influences SP.*

**Hypothesis** **4.**  *OC significantly and positively influences SP.*

**Hypothesis** **5.**  *OC significantly and positively influences SMO.*

**Figure 1:**
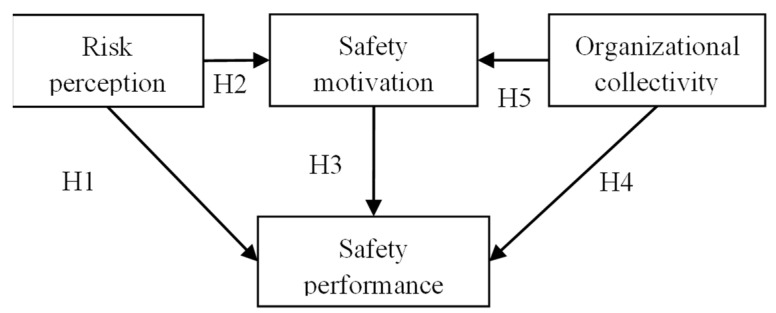
Integrated model of the influence on SP with hypothesized safety constructs.

The framework of the hypothesized research model is presented in Figure 1.

The mediating effect of SMO was hypothesized considering the transition role it plays among risk perception, organizational collectivity, and safety performance. On the one hand, the degree of SMO is influenced by individual awareness towards risks, which further motivates the workers’ willingness for obedience and compliance with safety instructions [40]. On the other hand, a harmonious atmosphere of a group facilitates the workers to form high willingness to participate in safety contribution and obey the requirement of their supervisor, thereby achieving great performance in work safety [41].

**Figure 2:**
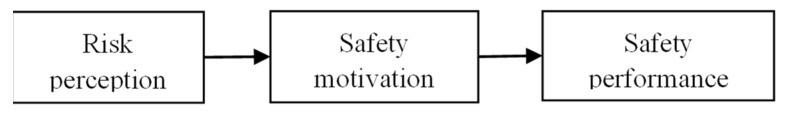
Hypothesized model of mediating effect of SMO between RPC and SP.

**Figure 3:**
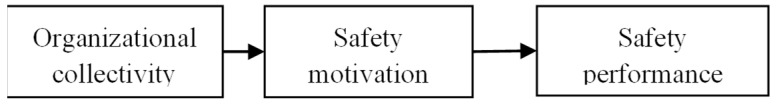
Hypothesized model of mediating effect of SMO between OC and SP.

The corresponding influencing models were depicted in Figure 2 and Figure 3.

**Hypothesis** **6.**  *SMO performs a significant and positive mediating effect between RPC and SP.*

**Hypothesis** **7.**  *SMO performs a significant and positive mediating effect between OC and SP.*

The regional comparison of the integrated SP models was conducted to provide targeted safety recommendations for the improvement of SP in Hong Kong and Mainland China.

### 2.6. Methodology

#### 2.6.1. Questionnaire Survey

The onsite questionnaire survey was performed from 1 February 2021 to 28 February 2022, which collected the participants’ responses about their agreement for 36 items measured by the Five-point Likert scale, in which “one” means “totally disagree” and “five” indicates “totally agree”. All respondents provided informed consent before participating in the survey. This research received the approval of the Ethics Sub-committee of Research Committee in China National Institute of Standardization and City University of Hong Kong. The questionnaire consists of four specific subscales which were adopted to measure the corresponding safety constructs. For SP, a scale developed by Naji et al. was applied, which contained six items to measure the two dimensions of SP [48]. OC was assessed using a 12-item scale, in which group cohesion was measured with 8 items designed by Kidwell et al. [49], and the collective efficiency was assessed using 4 items developed by Jex and Bliese [50]. A five-item scale designed by Vinodkumar and Bhasi was employed to quantify SMO of construction workers [33]. For RPC measurement, Man et al. has developed a 13-item scale, which was employed in the present study [18].

#### 2.6.2. Data Analysis

Data collected from the two regions were assessed using SPSS 24 for statistical analysis and Table 1 presents all the necessary evaluating indicators for analytic verification.

In addition, SEM was performed in AMOS 24 to assess the influence mechanism among safety constructs in both regions [55]. The study also applied Bootstrapping method in Amos 24, for testing the mediating effect of the social–cognitive constructs [56]. Moreover, the test of invariance routine was carried out in AMOS 24 to identify the performing distinction of the influence mechanism between the regions [57].

## 3. Results

### 3.1. Demographic Information

Table 2 shows the demographic profile of 377 respondents, among which the majority were male workers (58.38% for Mainland China and 81.11% for Hong Kong). The largest proportion of Mainland Chinese respondents were between 30 and 40 years old (46.19%), attained junior high school education (51.27%), worked in the construction industry for four to six years (34.52%), and had 46–50 working hours per week (43.15%). The major proportion of Hong Kong respondents had an average age between 40 and 50 (40.00%), worked for 13–15 years (41.11%), had a high school diploma (36.67%), and worked 46–50 h each week (26.67%). The job titles of the respondents in the two regions included quality inspector, safety inspector, project manager, constructor, and technician.

### 3.2. Validity and Reliability Tests

The reliability was tested, and the results are depicted in Table 3 and Table 4 Cronbach’s alpha of each safety construct was higher than 0.7, which presents the acceptability of the consistency reliability. Given the reasonable results of composite reliability (>0.7), AVE values (>0.5), and factor loadings (>0.7) for both Mainland China and Hong Kong, the convergent validity was verified to be acceptable [52]. Moreover, Table 5 verifies the acceptability of the discriminant validity for each safety construct by revealing that the largest correlation between one construct and others was less than its square root of AVE [53].

For the results of CFA, the Mainland China model shows the following coefficients: TLI = 0.958, RMSEA = 0.053, CFI = 0.968, χ^2^/df = 2.76, and SRMR = 0.015. These values clarify an outstanding fitness degree of the measurement model to the questionnaire data. For Hong Kong, the model fit results also indicate the high quality of fitness between the data and the measuring model (TLI = 0.912, χ^2^/df = 3.87, RMSEA = 0.075, CFI = 0.925, and SRMR = 0.047).

### 3.3. Structural Equation Modeling (SEM)

Two versions of the structural equation models were analyzed and compared in terms of Mainland China and Hong Kong to test the mechanism of direct and indirect influences among safety constructs on SP. The indexes of goodness-of-fit listed in Table 6 demonstrate that both models exhibit good fitness to corresponding data, as all the indicators met the relevant standards of model fit.

The significant regional distinctions of the research model performance were identified using the test of invariance routine. Table 7 shows the examined differences of goodness-of-fit indexes, degree of freedom, non-normed fit index, and comparative fit index, which reveals significant regional distinctions between SEMs in terms of the influence mechanism of SP [57].

**Figure 4:**
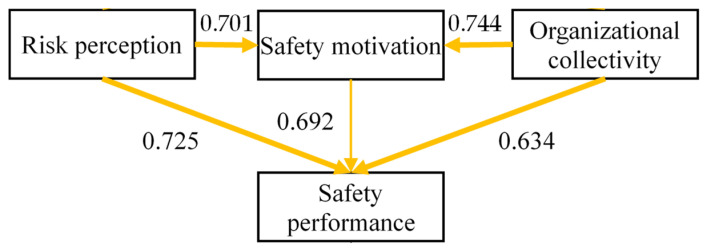
Structural equation modeling of SP in Hong Kong.

**Figure 5:**
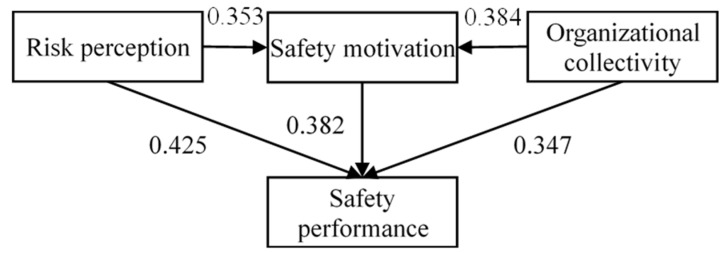
Structural equation modeling of SP in Mainland China.

Path coefficients were compared to further clarify the regional similarities and distinctions of every influence path. The two versions of SEMs are shown in Figure 4 and Figure 5. The influential intensity among safety constructs was compared between regions. With regard to the SP models, the results show that the influence mechanisms on SP in both the individual and organizational levels were stronger in Hong Kong (*p* < 0.005 for RPC, SMO, and OC), compared to those in Mainland China (*p* < 0.01 for PRC, SMO, and OC). Table 8 presents the results of the path coefficients and their corresponding significance levels. The width of the arrows in the figures reflects the intensity of the influences. Stronger effects between regions were highlighted in orange to emphasize the differences of the influence mechanism in the research model (see Figure 4).

### 3.4. Tests of Mediating Effects

The study further measured the mediating effects among safety constructs by using Bootstrapping in Amos 24 to verify and compare the influence mechanism of SP between regions [58]. As depicted in Table 9, the mediating effect of SMO was verified to be significant in two regions which played a role in linking two levels of SP models together. Particularly, the corresponding two effects were stronger in Hong Kong (*p* < 0.005) than in Mainland China (*p* < 0.01).

In addition, the direct, indirect, and total effects of the two models were concluded in Table 10 and Table 11. No indirect effects were observed between RPC and SMO, between OC and SMO, and between SMO and SP, which indicated that no mediating factor existed between the corresponding constructs. The influence mechanisms of SP models in both the individual and organizational levels were stronger in Hong Kong, since the direct effects of RPC and OC on SMO and SP were stronger in Hong Kong than in Mainland China. In addition, the influence of SMO on SP was greater in Hong Kong compared with its Mainland counterpart.

After verifying the effects of the safety constructs in the integrated models, the hypotheses were validated according to the results of data analysis. All the hypotheses were acceptable in both regions given the significance of the intercorrelation in the model (see Table 12).

## 4. Discussion

### 4.1. Cognitional Influence on Individual Perspective

The results present a significant and positive effect of individual risk perception towards safety performance of construction workers in both regions, though the corresponding influence in Hong Kong is proved to be stronger (*p* < 0.005) compared with that of Mainland counterparts (*p* < 0.01). Therefore, the authors suggest improving the level of risk perception of the construction workers as an alternative solution of safety performance promotion. For the regional difference between Hong Kong and China, the findings can echo the high rate of education level of Hong Kong construction workers, since the safety knowledge gained from the education help workers recognize and perceive the potential risks in motivated ways during construction work [59,60]. This further explains the stronger influential role and greater significance of RPC in individual SP mechanism of Hong Kong [61]. Accordingly, safety education and cultivation of workers’ conscientiousness are recommended for the construction industry in Mainland China, such as holding the training for personnel safety [62] or conducting metacognitive techniques to motivate the cognitional capability towards construction work safety [63].

### 4.2. Collectivistic Influence on Organizational Perspective

The influence of organizational collectivity is quantified to be positive and significant towards personnel’s safety performance, which is also verified to be greater in Hong Kong with a superior significance level (*p* < 0.005) in contrast to the Mainland model (*p* < 0.01). The findings highlight the feasibility of improving group cohesion and collective efficiency to achieve safety performance enhancement. For the former, concerned authorities should hold group therapies to increase the unity and cohesion of the organization, such as team-building activities which unify the goals and values of the members, thereby achieving group orientation of cohesion and collaboration [25]. For the latter, collective efficiency should be promoted by integrated workforce training across different divisions and departments so as to support the individuals effectively applying their capabilities and participating in organizational safety contributions, by motivating their confidence and overall efforts [64,65]. The regional difference of OC impact can be attributed to distinct organizational cultures and leadership styles in the two regions. According to Sheer, paternalistic leadership, which usually has low high convergent validity and fails to hold as a cohesive construct, is considered predominant in Mainland Chinese organizations, compared with that in Hong Kong and Taiwan [66].

### 4.3. Integration Role of Safety Motivation

The present study reveals that SMO played an important role in integrating the SP models in both the individual and organizational influence mechanisms. The results pointed out the transitional role of SMO in linking the two levels of SP model together as it verified the positive mediating effects of SMO with not only PRC, but also OC. This can be further explained by the theories of cognitional and behavioral motivation [67]. On the one hand, SMO is strongly determined by cohesion and collectiveness in organization, since the high collectivity and cohesion in workgroups foster a good communication environment of the coworkers and motivate their sharing and participation in safety contribution [68,69]. The high quality of organizational collectivity also stimulates the willingness of workers to obey the requirement of supervisors, therefore promoting the safety compliance of the employee [70]. On the other hand, the perceptions of workplace risks are verified to affect workers’ motivation and willingness to act safely first and then promote their safety behavior [12,71]. Workers with a higher cognitional level related to the safety and risk at work will be highly alert and attentive on finding out what they should do to ensure their personal safety and improve their risk-resistance, thus motivating themselves to obey the safety rules and participate in safety training [72,73].

The study further compared the effect of SMO between the two regions; the mediating role of SMO was proved to be stronger in sequential mediating influence together with individual perception and organizational collectivity in Hong Kong (*p* < 0.005). Therefore, considering the poor effect in Mainland China, motivational training with multiple methods (e.g., relaxation training, contingency management, cognitive restructuring, and mindfulness) in terms of cognitive and behavioral aspects should be applied in Mainland China to reduce the unsafe performance of construction workers by motivating their conscientiousness, long-term abstinence, and regulation observation [74,75]. Safety targets should be set by supervisors regularly and encouraged at the individual (e.g., helping 10 co-workers per month) and organizational levels (e.g., zero personal injuries and casualties in a working group), thereby achieving perceptional motivation in Mainland China [63,76].

## 5. Conclusions

This study was conducted to identify and compare the influence mechanisms on safety performance of construction workers between regions in both individual and organizational levels, by integrating individual risk perception and organizational collectivity together with safety motivation. Data were collected from 377 construction workers (197 from Mainland China and 180 from Hong Kong) through an on-site questionnaire survey. The findings contribute to both the theory underlying those influence mechanisms and industrial practices, which further reveal the scientific value and the applicability of the research.

### 5.1. Theoretical and Practical Contributions

The study provided theoretical implications toward occupational safety in the construction industry. Specifically, the present research quantitatively examined the influence mechanism on safety performance of construction workers in both individual and organizational levels, by combining two types of safety performance mechanisms linked by safety motivation. Scientific values of the study are therefore revealed, as the verified social–cognitive model of construction personnel can help assess the intercorrelation between social psychological factors and behavioral performance towards occupational safety. The regional similarities and distinctions were revealed after analyzing and comparing the direct, indirect, and mediating influence in the SEMs between Hong Kong and Mainland China.

The findings also provided important practical implications, particularly for construction safety management and relevant concerned authorities. The verified direct and indirect effects of safety constructs in a multilevel research model can assist construction management and concerned authorities in properly designing work for personnel safety improvement in Mainland China and Hong Kong, thereby reducing the number of construction accidents and fatalities in both regions. The research achievements contain high applicability and feasibility for practical use in industries. For instance, given the lower significance of the individual and organizational influence mechanisms on safety performance, the metacognitive techniques and safety promotional programs should be conducted in Mainland China with the aims of safety education, stimulating motivation, building conscientiousness, and improving organizational collectivity.

### 5.2. Research Limitations and Further Directions

The findings of the present study should be interpreted with some methodological limitations in mind. First, distributing hard copies of questionnaires among workers in construction sites in person was difficult due to the COVID-19 epidemic. Thus, the survey had to be suspended and delayed sometimes. Online platforms are therefore recommended for questionnaire distribution in further studies, as the data can be collected more conveniently through internet channels. Second, as a cross-sectional survey, this work only collected the questionnaire data within a short period instead of the overall process, which may only reveal a snapshot of the current situation of the influence mechanism of safety constructs on safety performance. Future research is suggested to conduct periodic surveys multiple times to obtain an understanding of the dynamic changes of safety performance for construction workers. Third, the measures applied in the research were mainly self-reported and drawn from the same source; the data collected from the scales were mainly reported by the respondents themselves. Future research is suggested to conduct the same study with the use of measures drawn from other sources, such as by inviting the leaders of the construction team to report on their subordinates’ performance of working safety.

## Figures and Tables

**Table 1:** Conclusion of statistical analysis and the evaluating criteria.

Assessments	Criteria	References
Consistency reliability	Cronbach’s alpha	[51]
Convergent validity	Values of average variance extracted (AVE) Composite reliability Factor loadings	[52]
Discriminant validity	Comparing the square root of AVE with the largest inter-construct correlations	[53]
Confirmatory factor analysis (CFA)	Tucker–Lewis Index (TLI) Chi-square divided by its degree of freedom (χ^2^/df) Root Mean Square Error of Approximation (RMSEA) Standardized Root Mean Square Residual (SRMR) Comparative Fit Index (CFI)	[54]

**Table 2:** Demographic information of respondents from Mainland China and Hong Kong.

Demographic Variable		Mainland China (197)		Hong Kong (180)	
		N	Percentage (%)	N	Percentage (%)
Gender	Male	115	58.38	146	81.11
	Female	82	41.62	34	18.89
Education background	Primary school or below	22	11.17	27	15.00
	Junior high school	101	51.27	42	23.33
	High school	46	23.35	66	36.67
	University or above	28	14.21	45	25.00
Age	20–30	83	42.13	36	20.00
	30–40	91	46.19	60	33.33
	40–50	14	7.11	72	40.00
	≥50	9	4.57	12	6.67
Weekly working hours	<40	4	1.02	10	5.56
	35–40	11	5.58	11	6.11
	41–45	49	24.87	46	25.56
	46–50	85	43.15	48	26.67
	51–55	41	20.81	31	16.11
	≥55	7	3.55	34	18.89
Years of working service	<3	39	19.80	19	10.56
	4–6	68	34.52	21	11.67
	7–9	44	22.34	38	21.11
	10–12	27	13.71	8	4.44
	13–15	11	5.58	74	41.11
	≥16	8	4.06	20	11.11
Job Title	Quality inspector	30	15.23	35	19.44
	Safety inspector	33	16.75	24	13.33
	Project manager	19	9.64	22	12.22
	Constructor	72	36.55	59	32.78
	Technician	43	21.83	40	22.22

**Table 3:** Statistical Analysis of the SC and SCB performances of construction workers.

Construct	Dimension	Item	Factor Loading (HK)	Factor Loading (MC)	Cronbach’s Alpha (HK)	Cronbach’s Alpha (MC)
RPC	Probability	1	0.855	0.863	0.804	0.821
		2	0.774	0.708		
		3	0.692	0.637		
	Seriousness	4	0.706	0.615		
		5	0.797	0.674		
		6	0.823	0.756		
		7	0.732	0.802		
	Worry and unsafety	8	0.694	0.721		
		9	0.673	0.652		
		10	0.705	0.726		
		11	0.827	0.804		
		12	0.882	0.755		
		13	0.762	0.653		
OC	Group cohesion	1	0.636	0.674	0.792	0.806
		2	0.689	0.712		
		3	0.742	0.763		
		4	0.753	0.835		
		5	0.824	0.867		
		6	0.777	0.814		
		7	0.795	0.684		
		8	0.852	0.863		
	Collective efficiency	9	0.691	0.788		
		10	0.752	0.687		
		11	0.823	0.842		
		12	0.884	0.798		
SMO		1	0.836	0.743	0.811	0.847
		2	0.792	0.785		
		3	0.654	0.726		
		4	0.799	0.821		
		5	0.823	0.673		
SP	SP1	1	0.809	0.884	0.859	0.755
		2	0.746	0.887		
		3	0.602	0.852		
	SP2	4	0.763	0.810		
		5	0.746	0.872		
		6	0.783	0.963		

**Table 4:** Results of AVE and composite reliability for data.

Safety Construct	Composite Reliability (HK)	Average Variance Extracted (HK)	Composite Reliability (MC)	Average Variance Extracted (MC)
OC	0.945	0.594	0.948	0.608
RPC	0.948	0.587	0.934	0.524
SMO	0.887	0.613	0.865	0.564
SP	0.880	0.554	0.953	0.772

**Table 5:** Confirmatory correlations of inter-factor among latent variables for Mainland China data.

	OC	RPC	SMO	SP
OC	0.780 (MC) 0.768(HK)			
RPC	0.586 ** (MC) 0.563 ** (HK)	0.724 (MC) 0.766 (HK)		
SMO	0.428 ** (MC) 0.572 ** (HK)	0.525 ** (MC) 0.492 ** (HK)	0.751 (MC) 0.783 (HK)	
SP	0.502 ** (MC) 0.485 ** (HK)	0.479 ** (MC) 0.453 ** (HK)	0.526 ** (MC) 0.465 ** (HK)	0.879 (MC) 0.744 (HK)

**: *p* < 0.01. significant.

**Table 6:** Model fit indexes for SEMs of Hong Kong and Mainland China.

	χ^2^/df	SRMR	TLI	CFI	RMSEA	GFI	AGFI	PGFI
Hong Kong Model	4.723	0.055	0.965	0.952	0.054	0.893	0.875	0.679
Mainland Model	4.357	0.043	0.953	0.941	0.062	0.913	0.896	0.682
Standard	≤5	≤0.08	≥0.9	≥0.9	≤0.08	≥0.5	≥0.8	≥0.5

**Table 7:** Comparisons for cross-regional SEMs between Hong Kong and Mainland China.

Comparison	ΔCFI	Δdf	$\Delta \mathrm{SB}-\chi^{2}$	ΔNNFI
Hong Kong vs. Mainland China	0.011 **	18 **	72.35 **	0.011 **

Note. CFI = comparative fit index; df = model degrees of freedom; SB*-*χ^2^ = Sattora–Bentler-scaled chi-square; NNFI = non-normed fit index; **: Significant correlation at 0.01 level.

**Table 8:** Significance of path impact among safety constructs in SEM.

Safety Construct	Sig (HK)	Sig (MC)	
RPC→SMO	0.701 ***	0.353 **	
RPC→SP	0.725 ***	0.425 **	
OC→SMO	0.744 ***	0.384 **	
OC→SP	0.634 ***	0.347 **	
SMO→SP	0.692 ***	0.382 **	

**: *p* < 0.01. ***: *p* < 0.005. ** and ***: significant.

**Table 9:** Mediating effect of safety constructs in the influence mechanism of safety performance in Hong Kong and Mainland China.

Influence Path	Mediating Effect	Bootstrapping				
		Percentile 95% CI		Bias-Corrected Percentile 95% CI		Sig (Two Tiled)
		Lower	Upper	Lower	Upper	
RPC→SMO→SP (HK)	0.579	0.527	0.631	0.527	0.631	***
OC→SMO→SP (HK)	0.611	0.509	0.713	0.510	0.714	***
RPC→SMO→SP (MC)	0.293	0.195	0.391	0.196	0.392	**
OC→SMO→SP (MC)	0.315	0.219	0.411	0.220	0.412	**

Note: Standardized estimation of 10,000 bootstrap samples, **: *p* < 0.01, ***: *p* < 0.005. ** and ***: significant.

**Table 10:** Total, direct, and indirect effects among safety constructs in Hong Kong.

		RPC	OC	SMO
SMO	Total	0.700 ***	0.744 ***	
	Direct	0.700 ***	0.744 ***	
	Indirect			
SP	Total	0.887 ****	0.886 ****	0.692 ***
	Direct	0.725 ***	0.634 ***	0.692 ***
	Indirect	0.162 *	0.252 *	

*: *p* < 0.05. ***: *p* < 0.005. * and ***: significant.

**Table 11:** Total, direct, and indirect effects among safety constructs in Mainland China.

		RPC	OC	SMO
SMO	Total	0.352 **	0.384 **	
	Direct	0.352 **	0.384 **	
	Indirect			
SP	Total	0.576 ***	0.538 ***	0.381 **
	Direct	0.422 **	0.347 **	0.381 **
	Indirect	0.154 *	0.191 *	

*: *p* < 0.05. **: *p* < 0.01. ***: *p* < 0.005. *, ** and ***: significant.

**Table 12:** Verification results of the hypotheses in safety performance mechanism.

No.	Hong Kong	Mainland China
Hypothesis 1	Accepted	Accepted
Hypothesis 2	Accepted	Accepted
Hypothesis 3	Accepted	Accepted
Hypothesis 4	Accepted	Accepted
Hypothesis 5	Accepted	Accepted
Hypothesis 6	Accepted	Accepted
Hypothesis 7	Accepted	Accepted

## Data Availability

The data presented in this study are available on request from the corresponding author. The data are not publicly available due to privacy and ethical consideration.
